# Supplementary figures and images for: Catch assemblages in the small-scale trap fishery with relation to hydrographic features of a tropical bay in the Gulf of Thailand
Source: PLoS One. 2023 Dec 21;18(12):e0296135. doi: 10.1371/journal.pone.0296135 (PMC10735179; doi:10.1371/journal.pone.0296135)

● measurement — surface — bottom

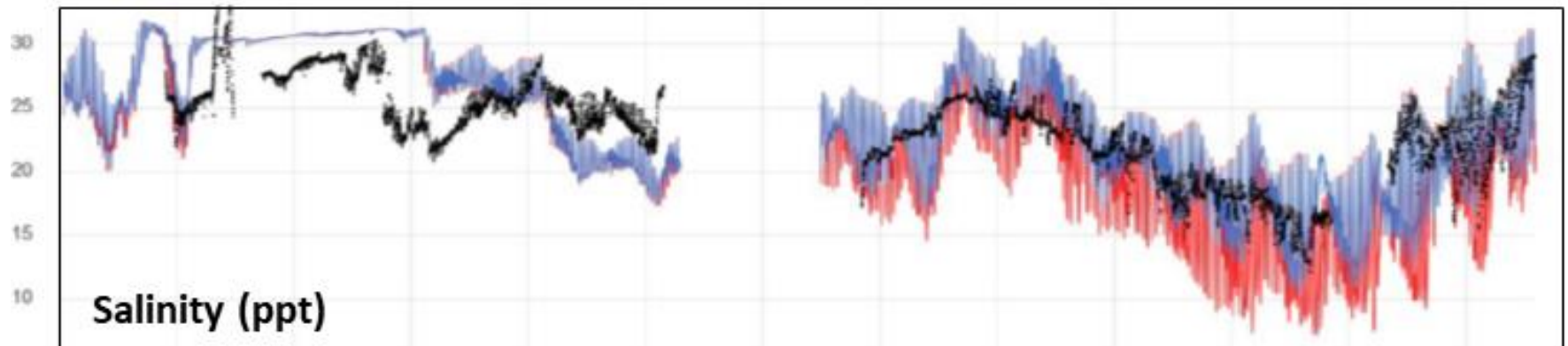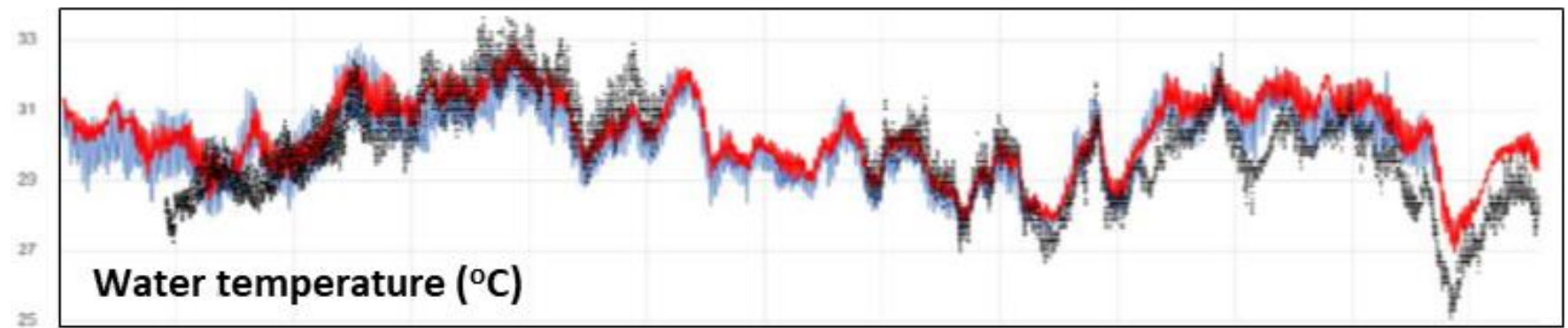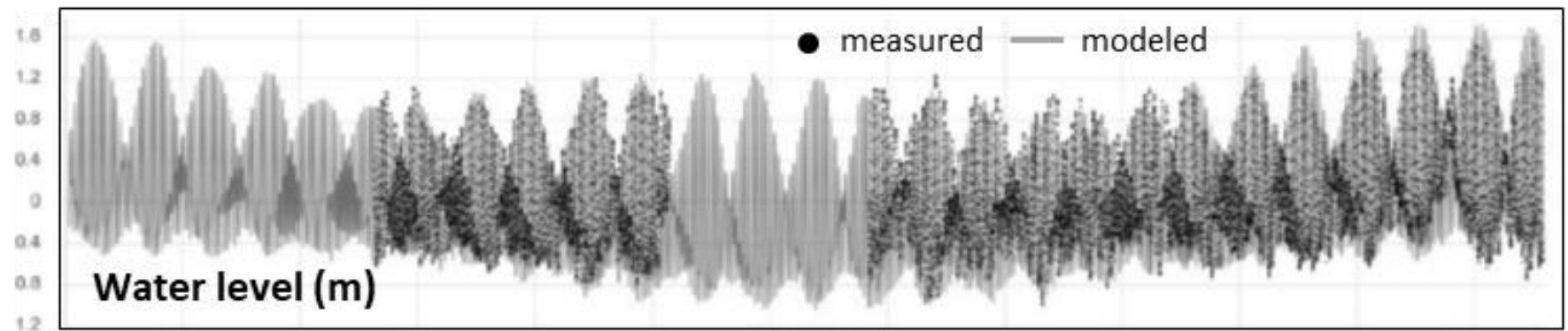

Dec-18 Jan-19 Feb-19 Mar-19 Apr-19 May-19 Jun-19 Jul-19 Aug-19 Sep-19 Oct-19 Nov-19 Dec-19

Supplement: S1 Fig — (PDF) [file pone.0296135.s001.pdf]

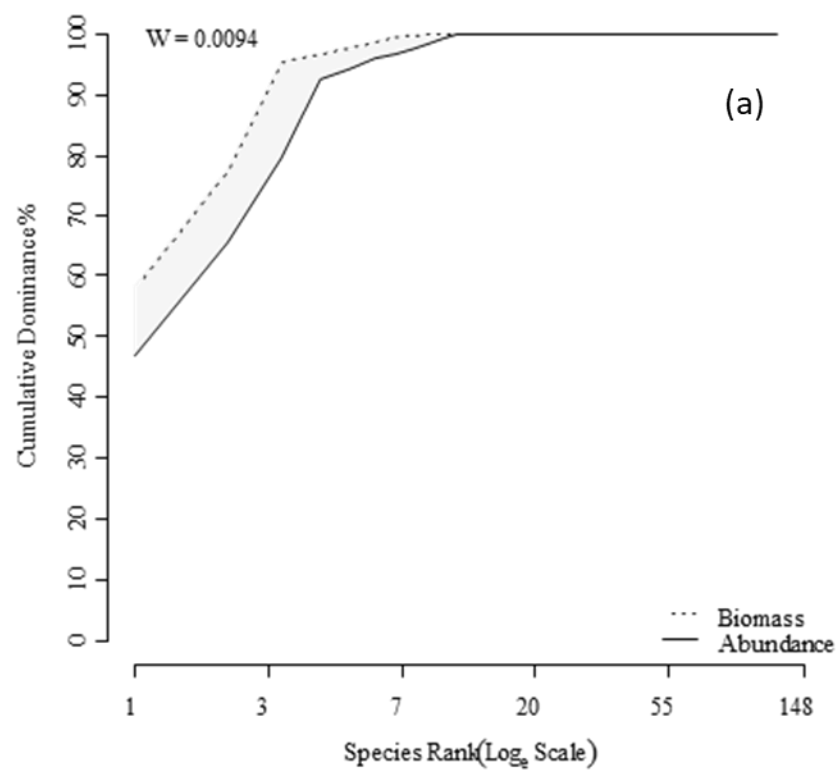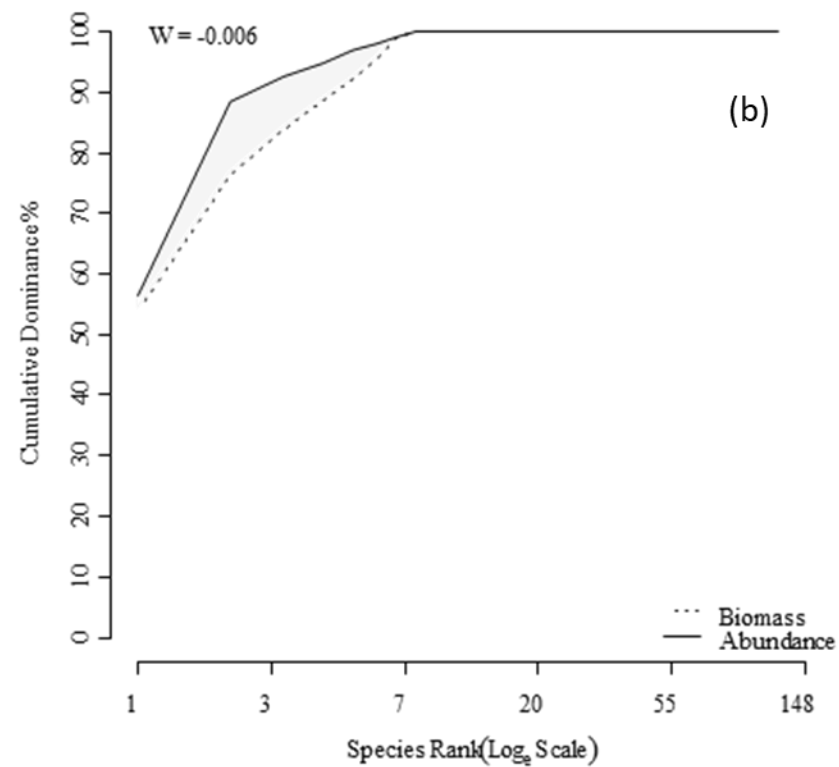

Supplement: S2 Fig — (a) example of ABC for undisturbed community with a positive sign W-statistic and (b) example of ABC for disturbed community with a negative sign W-statistic. (PDF) [file pone.0296135.s002.pdf]

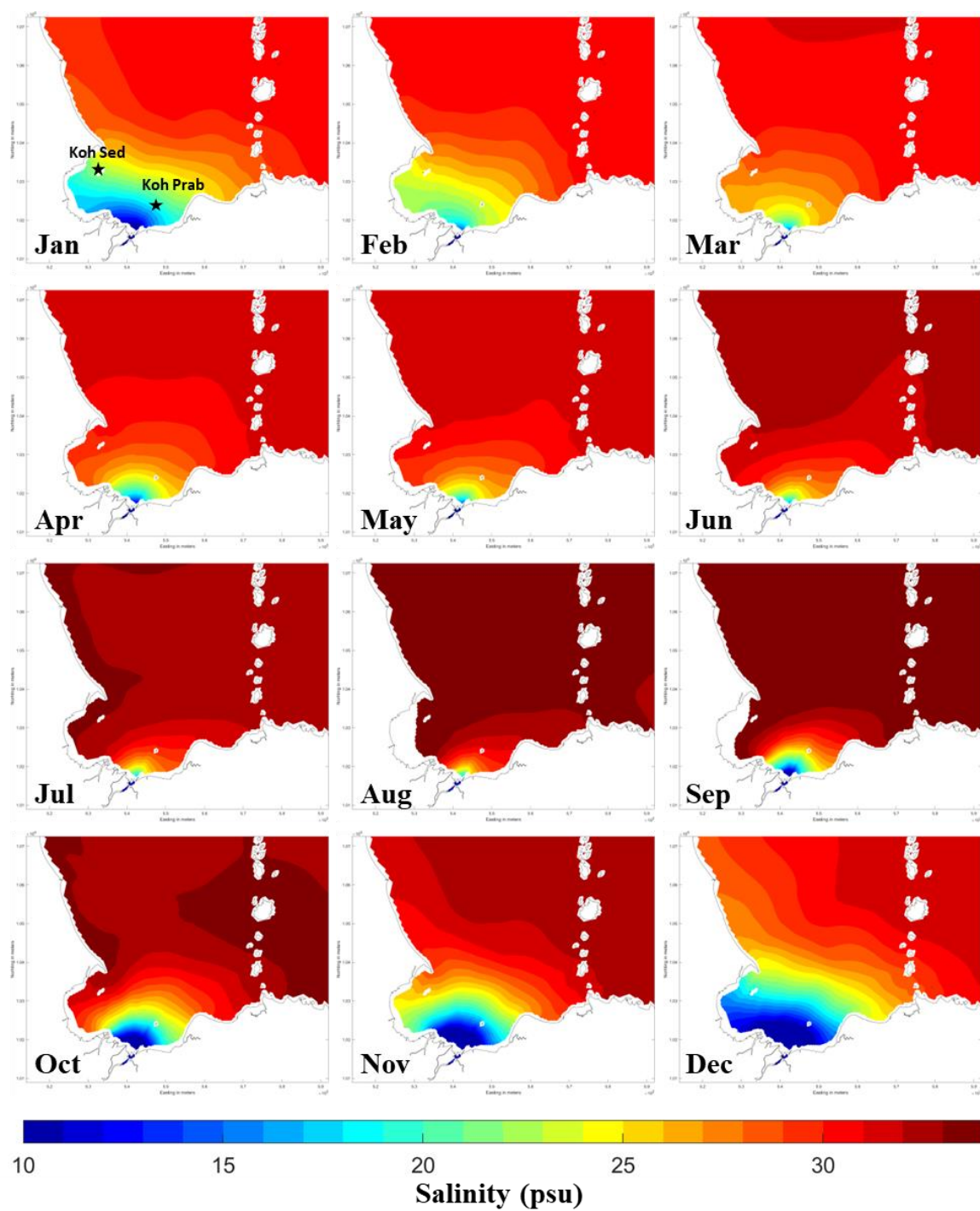

Supplement: S3 Fig — (PDF) [file pone.0296135.s003.pdf]

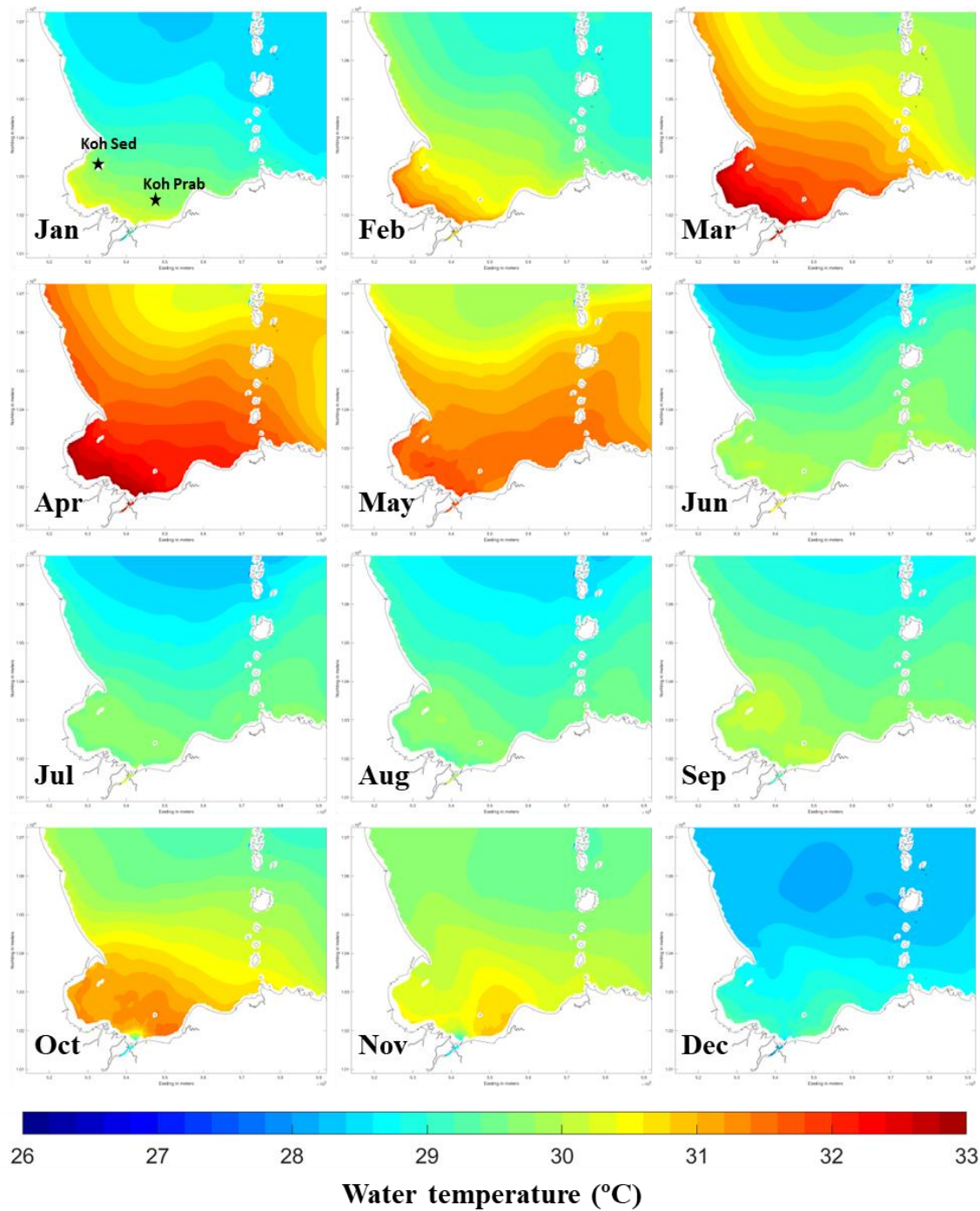

Supplement: S4 Fig — (PDF) [file pone.0296135.s004.pdf]

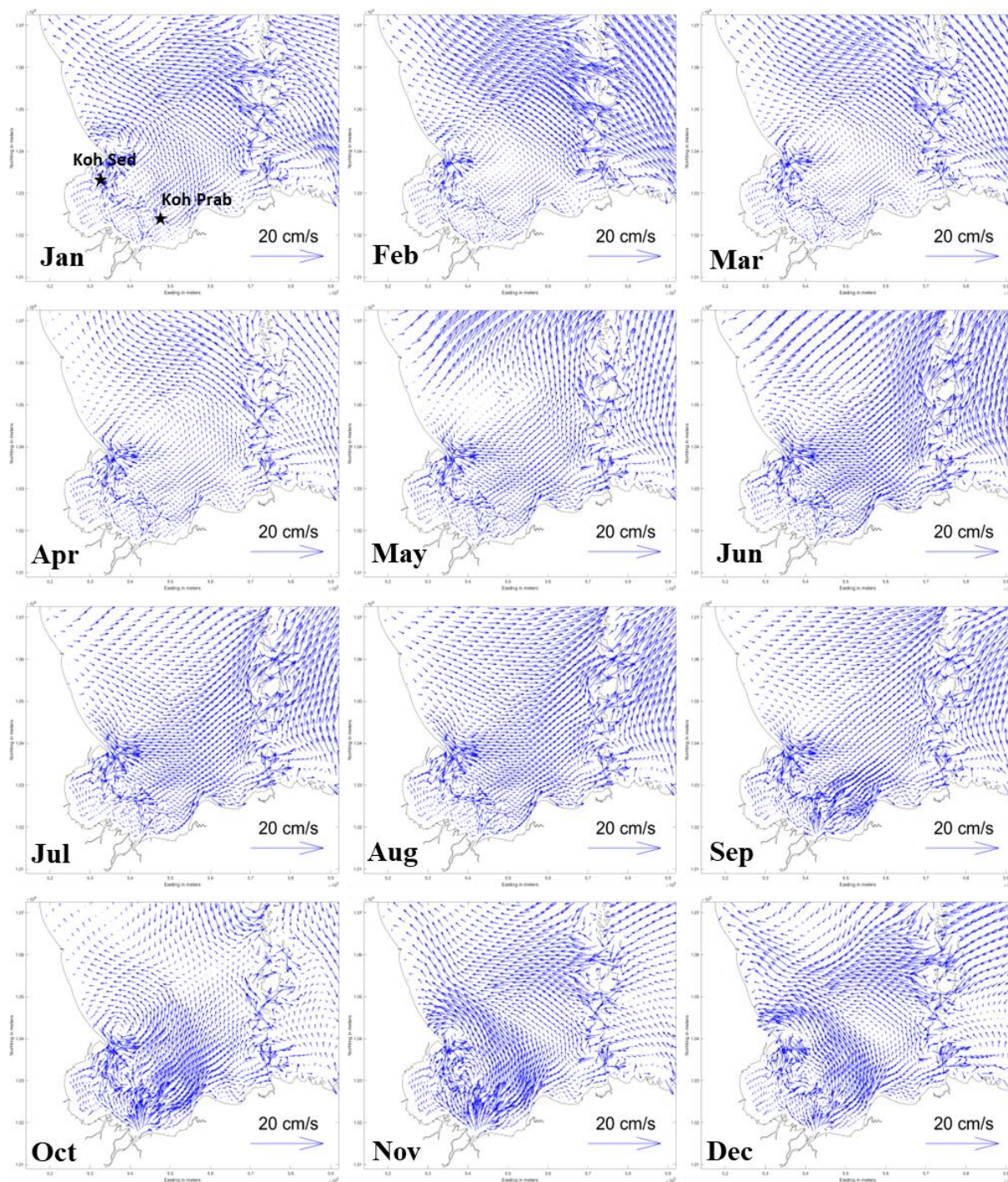

Supplement: S5 Fig — (PDF) [file pone.0296135.s005.pdf]

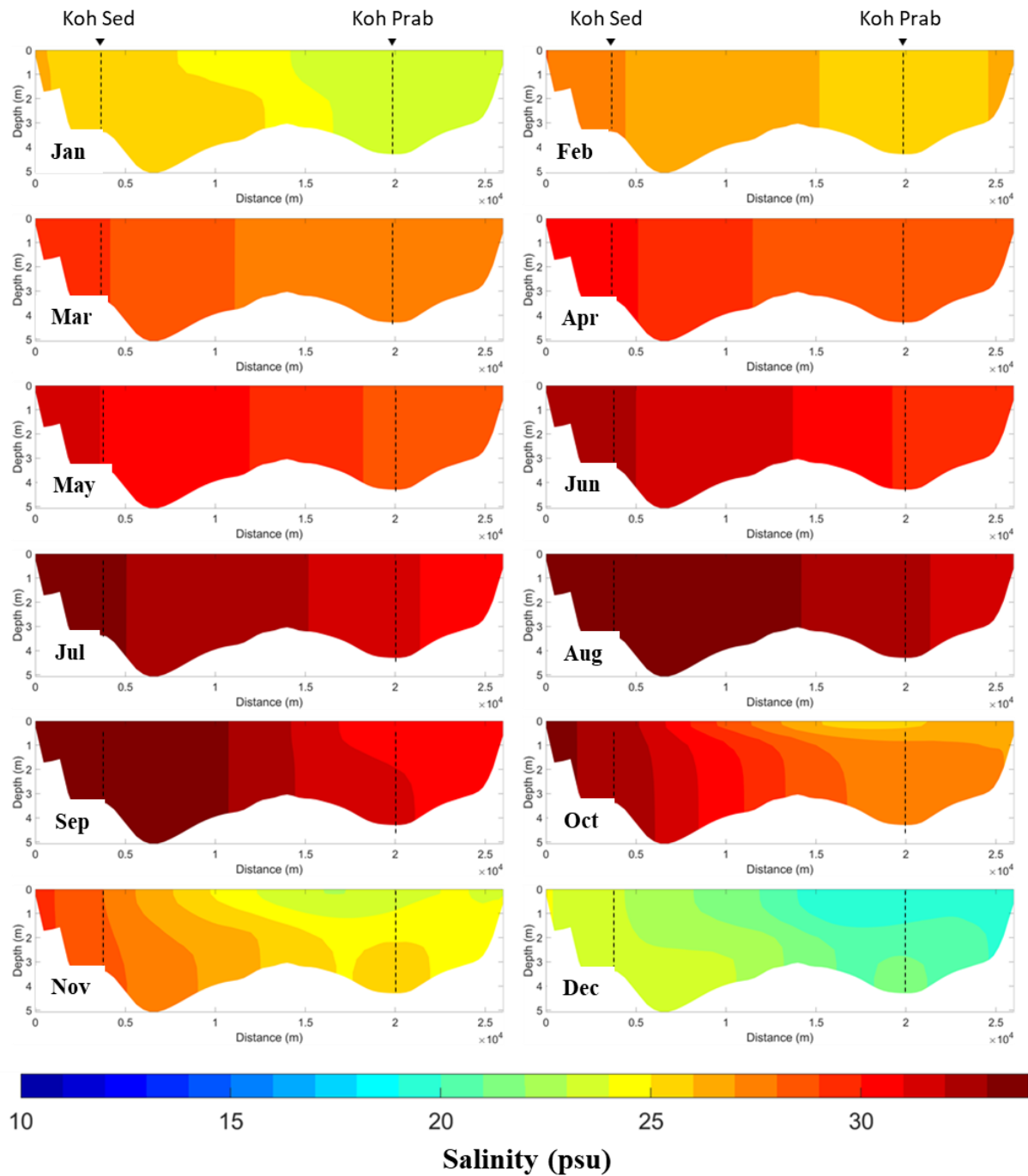

Supplement: S6 Fig — (PDF) [file pone.0296135.s006.pdf]

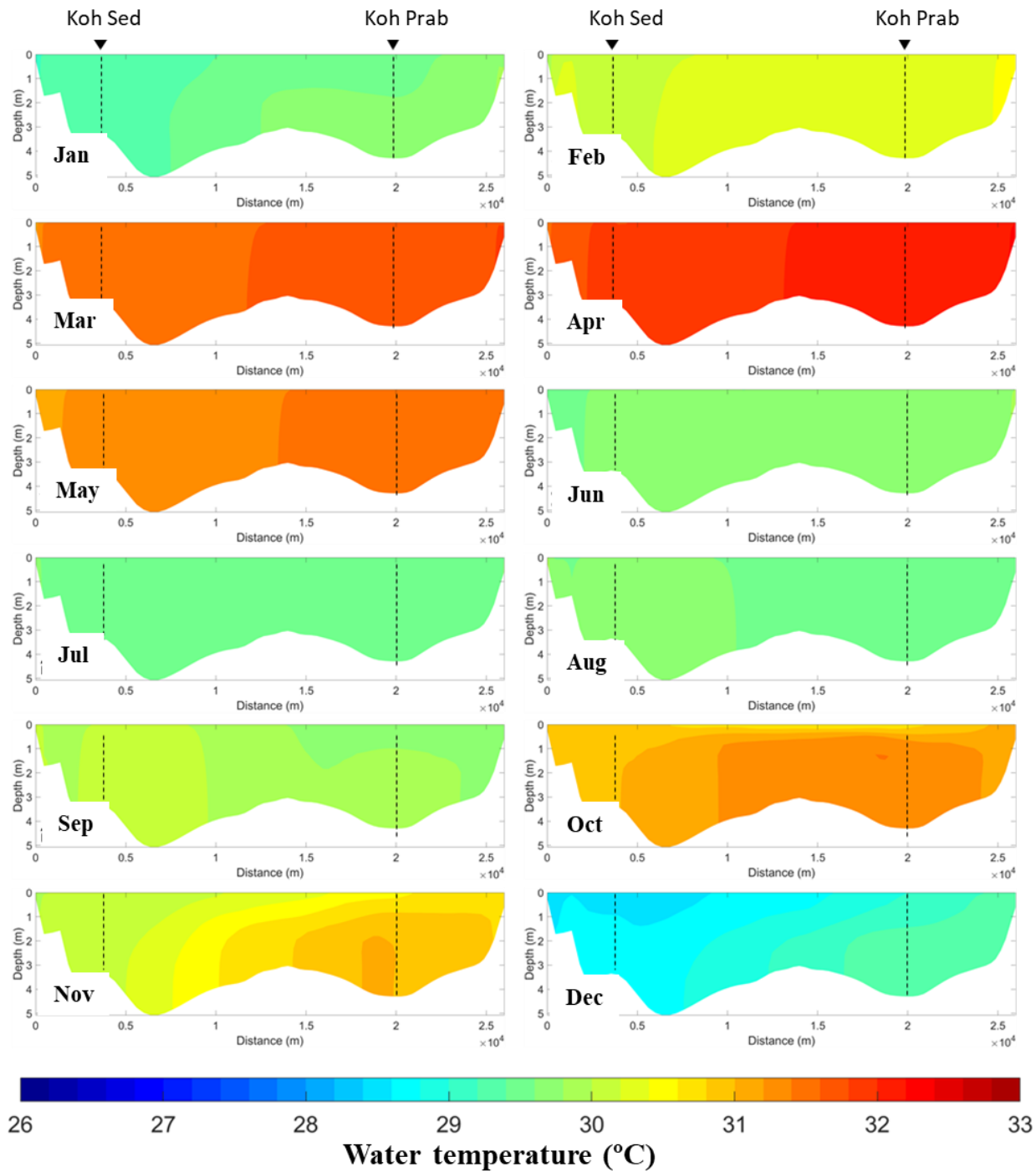

Supplement: S7 Fig — (PDF) [file pone.0296135.s007.pdf]

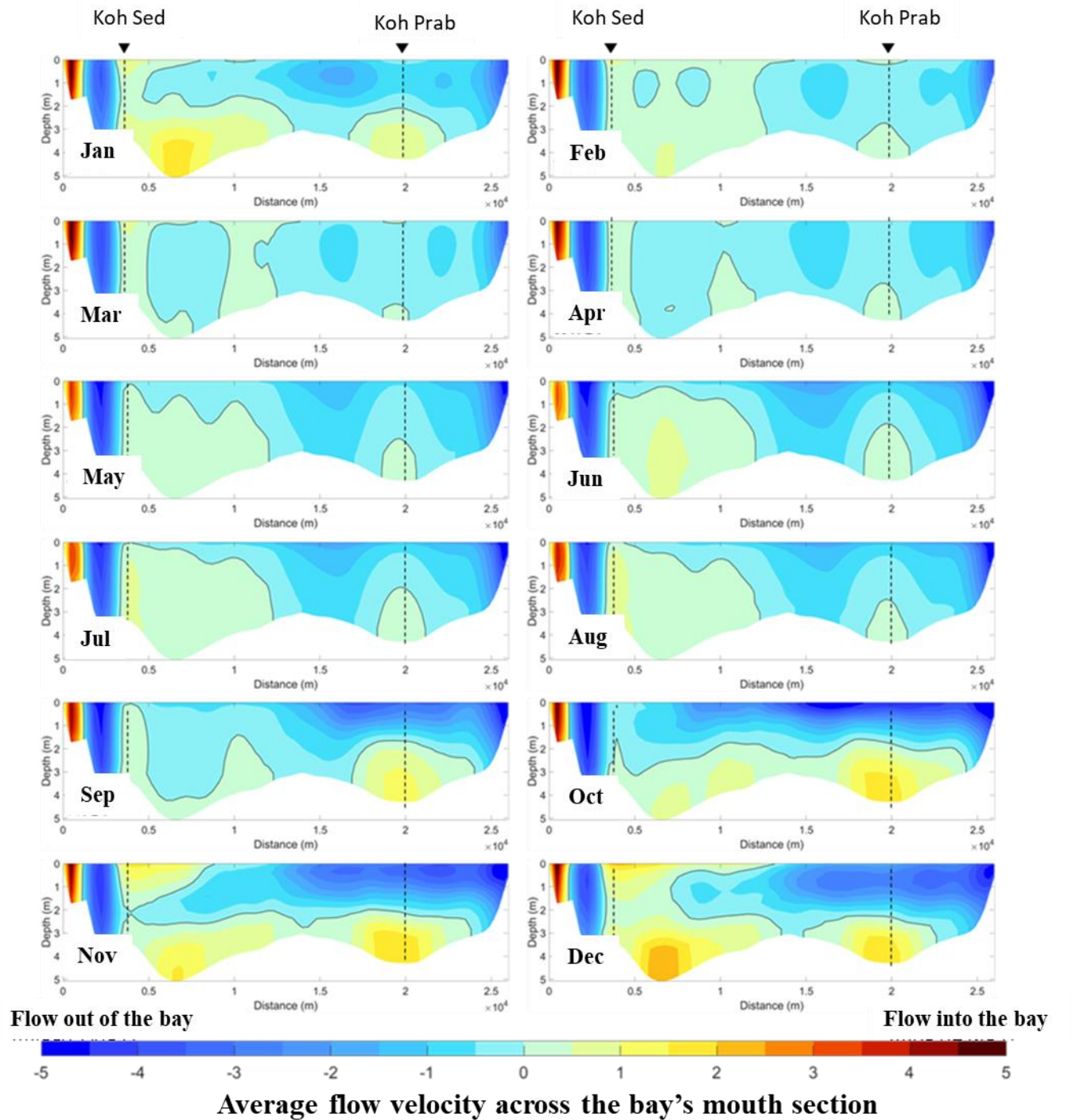

Supplement: S8 Fig — (PDF) [file pone.0296135.s008.pdf]
