# Supplementary material for: Catch assemblages in the small-scale trap fishery with relation to hydrographic features of a tropical bay in the Gulf of Thailand
Source: PLoS One. 2023 Dec 21;18(12):e0296135. doi: 10.1371/journal.pone.0296135 (PMC10735179; doi:10.1371/journal.pone.0296135)
Supplement: Attachment — Submitted filename: Response to Reviewers (PONE-D-23-26740).docx [file pone.0296135.s010.docx]

**Response / Rebuttal letter**

**PONE-D-23-26740:** Catch assemblages in the small-scale trap fishery with relation to hydrographic features of a tropical bay in the Gulf of Thailand

**Response to reviewers**

| **Comments** | **Responses** |
| --- | --- |
| **Reviewer #1** |  |
| General comments | Thank you very much for the general comment by Reviewer #1. |
| There is no specific comment made by Reviewer #1 |  |
| **Reviewer #2** |  |
| General comments | Thank you very much for the general comment by Reviewer #2. I |
| In figure 1, the 14th sampling site is not indicated. | Figure 1 was already revised and made correction |
| Line 124: the name of cyclone is Pabuk | The name of the cyclone in text was already corrected. |
| Line 214: correct the sampling year | The year in caption of Table 1 was already corrected, i.e., 2018 |

**Note:** The specific comments in text, i.e., Lines 124 and 214 are yellow highlighted

**Response to journal requirements**

1. Please ensure that your manuscript meets PLOS ONE's style requirements, including those for file naming.

**Response:** We confirm that the manuscript is followed PLOS ONE's style requirements

2. Please complete your Competing Interests on the online submission form to state any Competing Interests.

**Response:** We did state "The authors have declared that no competing interests exist."

3. Data availability, if there are no restrictions, please upload the minimal anonymized data set necessary to replicate your study findings as either Supporting Information files or to a stable, public repository and provide us with the relevant URLs, DOIs, or accession numbers.

**Response:** We did upload the minimal anonymized data set as Supporting Information file

4. PLOS requires an ORCID iD for the corresponding author in Editorial Manager

**Response:** The ORCID number of the corresponding author (0000-0002-7179-7913) is updated in Editorial Manager

5. Please upload a new copy of Figure 7 as the detail is not clear.

**Response:** A new copy of Figure 7 is uploaded

6. We note that [Figures 1, 6 and S3-8] in your submission contain [map/satellite] images which may be copyrighted.

**Response:** We confirm that all figures are our original artwork.

- Figure 1 was made by using ArcGIS11.1 (ESRI customer number 161475), The district boundary and river channels was downloaded from <https://csuwan.weebly.com/360436343623360936603650362736213604--download.html>
- For Figures 6 and 7 and Figures S3 to S8, we confirm that they are all original Figures created from data resulted from our studies. We applied self-coding MATLAB scripts to plot data from original setup Delft3D-FLOW model. Both the MATLAB license no. 40904944 program and Delft3D-FLOW program are under academic license of Kasetsart University – Thailand.

7. Please review your reference list to ensure that it is complete and correct.

**Response:** We did check the references and there is no retracted paper is cited.

Also, May I correct my affiliation in the Title and Authors page from

4. Faculty of Agriculture, Ubon Ratchathani University, Thailand to

4. Faculty of Agriculture, Ubon Ratchathani University, Ubon Ratchathani, Thailand

**END OF RESPONSE**
